# Synergistic Effects and Mechanisms of Budesonide in Combination with Fluconazole against Resistant *Candida albicans*

**DOI:** 10.1371/journal.pone.0168936

**Published:** 2016-12-22

**Authors:** Xiuyun Li, Cuixiang Yu, Xin Huang, Shujuan Sun

**Affiliations:** 1 School of Pharmaceutical Sciences, Shandong University, Jinan, Shandong Province, People’s Republic of China; 2 Respiration Medicine, Qianfoshan Hospital Affiliated to Shandong University, Jinan, Shandong Province, People’s Republic of China; 3 Pharmaceutical Department, Qianfoshan Hospital Affiliated to Shandong University, Jinan, Shandong Province, People’s Republic of China; Institute of Microbiology, SWITZERLAND

## Abstract

*Candida albicans* is an important opportunistic pathogen, causing both superficial mucosal infections and life-threatening systemic diseases in the clinic. The emergence of drug resistance in *Candida albicans* has become a noteworthy phenomenon due to the extensive use of antifungal agents and the development of biofilms. This study showed that budesonide potentiates the antifungal effect of fluconazole against fluconazole-resistant *Candida albicans* strains both *in vitro* and *in vivo*. In addition, our results demonstrated, for the first time, that the combination of fluconazole and budesonide can reverse the resistance of *Candida albicans* by inhibiting the function of drug transporters, reducing the formation of biofilms, promoting apoptosis and inhibiting the activity of extracellular phospholipases. This is the first study implicating the effects and mechanisms of budesonide against *Candida albicans* alone or in combination with fluconazole, which may ultimately lead to the identification of new potential antifungal targets.

## 1. Introduction

Globally, *Candida albicans* (*C*. *albicans*) is the leading human fungal pathogen, and the incidence of invasive fungal infections caused by *C*. *albicans* is rising, along with an increasing mortality rate [1–3]. Fluconazole (FLC), one of the triazole antifungal agents, is the most widely used antifungal agents to treat candidiasis infections due to its high bioavailability and low toxicity [4–6]. However, the excessive and indiscriminate use of FLC in the clinic has led to the emergence of drug resistance in *C*. *albicans* [7–9]. Biofilms formed on medical devices not only provide protection from environmental stress for *C*. *albicans* but also enhance the resistance of *C*. *albicans* to antifungal agents by up to 1000-fold greater than that needed for the treatment of its planktonic counterparts [10–12]. Therefore, it is necessary to develop new antifungal strategies that can eliminate the phenomenon of drug resistance in *C*. *albicans*. The discovery of new antifungal agents is a complicated and long-term process [13, 14]. To develop new approaches for the treatment of invasive fungal infections, combination therapy may be an ideal approach. It could significantly improve the antifungal efficacy of a drug compared to each drug used alone and reduce adverse side effects from the drugs used. In recent years, efforts have been made to overcome the resistance of *C*. *albicans* by using drug combinations [15, 16].

Glucocorticoids usually are not used in general clinical anti-infection therapy in consideration of their immunosuppressive actions, and they are only used in severe infections and septic shock for their strong anti-inflammatory effects. We found, however, that budesonide (BUD), an inhaled corticosteroid, can enhance the antifungal effect of FLC against resistant *C*. *albicans in vitro*.

In the present report, we first investigated the *in vitro* effects of BUD combined with FLC against resistant *C*. *albicans* and susceptible *C*. *albicans* by a microdilution checkerboard method. *Galleria mellonella* (*G*. *mellonella*), a new fungal infection model, was used to study the effects of the combined treatment on *C*. *albicans in vivo*. The results *in vitro* and *in vivo* strongly revealed that the combination of FLC and BUD has synergistic effects against resistant *C*. *albicans*. Then, we further investigated the molecular mechanisms of BUD for enhancing the susceptibility of resistant *C*. *albicans* to FLC by assaying its impact on the function of drug transporters, biofilm formation, the induction of apoptosis and the activity of extracellular phospholipases, as well as assaying the expression levels of the genes related to these physiological processes in *C*. *albicans*. Our results demonstrated that the combination of FLC and BUD can reverse the resistance of *C*. *albicans* by inhibiting the function of drug transporters, reducing the formation of biofilms, promoting apoptosis and inhibiting the activity of extracellular phospholipases. We believe that researchers in the field of *C*. *albicans* may benefit from the conclusions of our studies.

## 2. Materials and Methods

### 2.1 Strains and growth medium

Two resistant *C*. *albicans* strains: *C*. *albicans* 10 (CA10) and *C*. *albicans* 16 (CA16) and two susceptible *C*. *albicans* strains: *C*. *albicans* 4 (CA4) and *C*. *albicans* 8 (CA8) were used, and *C*. *albicans* ATCC10231 was used as a quality control. CA4, CA8, CA10 and CA16 were provided by clinical laboratory in Qianfoshan Hospital, Jinan, China. ATCC10231 strain was generously provided by the Department of Pharmacology, School of Pharmaceutical Sciences, Shandong University, Jinan, Shandong Province, Jinan, China. All strains were refreshed from frozen storage at -80°C and subcultured on yeast–peptone–dextrose (YPD) solid medium (1% yeast extract, 2% peptone, 2% glucose and 2% agar) overnight at 35°C at least twice. Before each experiment, all strains were inoculated in YPD liquid medium (1% yeast extract, 2% peptone and 2% glucose) for 24 h at 35°C.

### 2.2 Drugs and *G*. *mellonella* larvae

All drugs (BUD, FLC and ampicillin) were purchased from Dalian Meilun Biotech Co., Ltd (Dalian, China). The stock solution of BUD (12800 μg/mL) was prepared in ethyl alcohol absolute. Stock solutions of FLC (2560 μg/mL) and ampicillin (2560 μg/mL) were prepared in distilled water. All stock solutions were stored at -20°C until use. *G*. *mellonella* larvae were purchased from Tianjin Huiyude Biotech Co., Ltd (Tianjin, China). Randomly chosen *G*. *mellonella* larvae (approximately 0.25 g) in the final instar larval stage were used in our experiments. *G*. *mellonella* larvae were cleaned with 70% ethanol and incubated in the dark at 37°C overnight prior to each experiment.

### 2.3. Determination of MICs of planktonic cells *in vitro*

Two resistant *C*. *albicans* strains (CA10 and CA16) and two susceptible *C*. *albicans* strains (CA4 and CA8) were used in this experiment. The antifungal effects of BUD in combination with FLC on *C*. *albicans* planktonic cells were evaluated using a checkerboard broth microdilution method in 96-well plates according to the protocol of the Clinical and Laboratory Standards Institute (CLSI, 2008 M27-A3). Drugs were added to the wells and serially diluted to final concentrations ranging from 0.125 μg/mL to 64 μg/mL for FLC and from 2 μg/mL to 128 μg/mL for BUD. 100μL *C*. *albicans* suspension at a final concentration of 2.5×10^3^ colony forming units (CFU)/mL was subsequently added to each well. A well containing 100 μL of *C*. *albicans* suspension and 100 μL of RPMI 1640 medium was included to serve as a drug-free control. The 12th column of wells on the plate was filled with only 200 μL of RPMI 1640 medium to act as negative background controls during subsequent analysis and quantification. Ninety-six-well plates were then incubated at 35°C for 24 h. A 2,3-bis-(2-methoxy-4-nitro-5- sulfophenyl)-2H-tetrazolium-5-carboxanilide (XTT) reduction assay was performed to determine the minimum inhibitory concentrations (MICs). The MICs are defined as the lowest antifungal concentrations at which there is an 80% (MIC_80_) reduction in XTT-colorimetric readings in comparison to the drug-free control. Colorimetric changes were measured at 492 nm with a microtiter plate reader (SpectraMax190, USA). All experiments were performed three times on different days.

### 2. 4. Determining the antifungal effects *in vivo*

#### *G*. *mellonella* survival assay

The *G*. *mellonella* infection model was performed as we described previously [16] with minor modifications, and the FLC-resistant isolate CA10 was used in this experiment. Briefly, 16 randomly chosen *G*. *mellonella* larvae were included in each group, and four groups were used in this experiment. *C*. *albicans* inoculum was suspended in ampicillin-PBS containing 2 μg/mL ampicillin to avoid bacterial interference. *C*. *albicans* inoculum at a concentration of 5×10^8^ CFU/mL was selected to monitor virulence. A 50-μL micro syringe (Shanghai Gaoge Biotech Co., Ltd. China) was used to inject 10 μL *C*. *albicans* inoculum (5×10^6^ CFU/larva) into the *G*. *mellonella* larval hemocoel via the last right proleg. After 2 h, *G*. *mellonella* larvae in four groups were injected with 10 μL PBS, FLC (160 μg/mL), BUD (160 μg/mL) or with a combination of FLC and BUD at the same concentrations. *G*. *mellonella* larvae injected with PBS were used as controls to control for the impact of physical trauma. After injection, *G*. *mellonella* larvae were incubated in sterile petri dishes at 37°C for 4 days, and larval death was monitored by visual inspection every day. A *G*. *mellonella* larva is considered dead when it displays no movement or proleg movement in response to touch [17]. Survival curves were analyzed by the Kaplan–Meier method using the Statistical Product and Service Solutions 19 software.

#### Fungal burden determination

The CFU counting method was used to determine the fungal burden of *G*. *mellonella* larvae infected with *C*. *albicans* CA10 [16]. *G*. *mellonella* larvae in four groups were treated with *C*. *albicans* inoculum and drugs as mentioned above. Three infected *G*. *mellonella* larvae were selected randomly from each group daily, washed with 70% ethanol and cut into small pieces with a scalpel. Pieces of each *G*. *mellonella* larva were put into a sterile tube containing 5 mL PBS-ampicillin and were mixed using a VORTEX GENIE 2 vibrator (Scientific Industries, USA). Then, different dilutions were made for each sample, and a 10-μL suspension from each dilution was placed on YPD solid medium dishes. The dishes were then incubated at 35°C for 24 h, and the CFU was counted. Graph Pad Prisma 5 software was used to analyze the CFU. All experiments were performed three times on different days.

#### Histology

Histology was used to characterize the development of the infection in *G*. *mellonella* larvae and to determine the effect of drugs on the *G*. *mellonella* larvae infected by *C*. *albicans* CA10 [18]. *G*. *mellonella* larvaein four groups were treated with *C*. *albicans* inoculum and drugs as mentioned above. One infected *G*. *mellonella* larva was randomly selected from each group after 2 days of infection, washed with 70% ethanol and cut into tissue sections (7 μm) using a freezing microtome (LEICA CM 1950, Germany). The tissue sections were then stained with Periodic Acid Schiff reagent (PAS) and observed under an OLYMPUS BX51 microscope using a 10×eyepiece and a 10×objective.

### 2. 5. Assaying the uptake and efflux of Rh6G

Rhodamine 6G (Rh6G) uptake and efflux in *C*. *albicans* CA10 cells were determined as previously described [15, 19, 20]. The cells were diluted to a final concentration of 1×10^5^ CFU/ mL with YPD liquid medium and incubated at 35°C. After 18–19 h, the cells were centrifuged, washed with glucose-free PBS (pH = 7.0) three times, and resuspended in glucose-free PBS to a concentration of 1×10^7^ CFU/mL. The cells were de-energized for 1–2 h in glucose-free PBS and then resuspended in glucose-free PBS for further use. For the Rh6G uptake experiment, Rh6G (Sigma, USA) and BUD were added to the above-mentioned cells at a final concentration of 10 μM and 128 μg/mL, respectively. A drug-free sample with Rh6G served as a control. After incubation at 35°C for 50 min, a 10 min of ice-water bath was used to stop cellular uptake of Rh6G, and then the extracellular Rh6G was removed using glucose-free PBS. The mean fluorescent intensity (MFI) of the remaining intracellular Rh6G was detected using a BD FACSAria Ⅱ flow cytometer (Becton Dickinson, USA) with excitation at 488 nm and emission at 530 nm. For the Rh6G efflux experiment, Rh6G was first added to the above-mentioned cells at a final concentration of 10 μM. After the incubation at 35°C for 50 min, a 10 min ice-water bath was used to stop Rh6G uptake into cells. Cells were then harvested, washed three times with glucose-free PBS to remove the extracellular Rh6G, and resuspended in PBS containing 5% glucose to initiate ATP-driven efflux. Meanwhile, BUD was added to the suspension at a concentration of 128 μg/mL. A drug-free sample with only Rh6G served as the control group. The MFI of the intracellular remaining Rh6G was detected every 30 min as mentioned above. Graph Pad Prisma 5 software was used to analyze the results. All experiments were performed three times on different days.

### 2. 6. Assaying the sessile MICs of *C*. *albicans* biofilms

The effects of BUD in combination with FLC against the biofilms of *C*. *albicans* CA10 were tested in 96-well plates as Ramage *et al*. described previously [21]. Briefly, each well in the plate was filled with 200 μL of *C*. *albicans* cells at a final concentration of 2.5×10^3^ CFU/mL, and the plates were incubated at 35°C for 4, 8, 12 or 24 h to obtain biofilms at different stages of maturation. The 12th column of wells on the plate was filled with 200 μL RPMI 1640 medium as a negative background control for subsequent analysis and quantification. After incubation, planktonic and nonadherent cells were removed, and the biofilms were washed with sterile PBS three times. Drugs were serially diluted to final concentrations ranging from 2 μg/mL to 1024 μg/mL for FLC or from 8 μg/mL to 512 μg/mL for BUD and added to the biofilm-coated wells. A drug-free well was included to serve as a positive control. The plates were incubated for an additional 24 h at 35°C. An XTT reduction assay was performed to determine the sessile minimum inhibitory concentrations (SMICs). The SMICs are defined as the lowest antifungal concentrations at which an 80% (SMIC_80_) reduction in XTT-colorimetric readings is found in comparison to the drug-free control. Colorimetric changes were measured at 492 nm with a microtiter plate reader (SpectraMax190, USA). All experiments were performedthree times on different days.

### 2. 7. Assaying the changes in reactive oxygen species

The endogenous reactive oxygen species (ROS) in *C*. *albicans* CA10 cells were measured using the dihydrorhodamine 123 (DHR 123, Sigma, USA) staining method [22]. Briefly, cells (1×10^6^ CFU/mL) were incubated in YPD liquid medium overnight at 35°C with drugs alone or in combination at the following final concentrations: FLC at 1 μg/mL; BUD at 128 μg/mL. A sample without the drug treatment was included as a control. After incubation, cells were centrifuged, washed with sterile PBS (pH = 7.0) three times, resuspended in sterile PBS to a concentration of 1×10^7^ CFU/mL and stained with DHR 123 (10 μM) in the dark at 35°C for 50 min. Finally, the MFI was detected using the BD FACSAria Ⅱ flow cytometer (Becton Dickinson, USA) with excitation at 488 nm and emission at 530 nm. Graph Pad Prisma 5 software was used to analyze the results. All experiments were performed three times on different days.

### 2. 8 Assaying phospholipase activity

The determination of extracellular phospholipase activity for *C*. *albicans* CA10 cells was performed according to the egg yolk agar method as previously described [23, 24] with a few modifications. Cells (1×10^6^ CFU/mL) were incubated in YPD liquid medium overnight at 35°C with treatment using drugs alone or in combination at the following final concentrations: FLC at 1 μg/mL; BUD at 128 μg/mL. Samples without drug treatment were included as controls. After incubation, a 10 μL suspension of each sample was deposited on plates containing the egg yolk agar medium (1% peptone, 3% glucose, 5% NaCl, 0.0006% CaCl2, 2% agar and 10% egg yolk). Plates were then incubated at 35°C for 48 h. The precipitation zone (Pz) value was calculated as follows [25]: Pz = $\frac{Diameter\;of\;colony}{Diameter\;of\;the\;colony\;plus\;precipitation\;zone}$. The Pz value was then used to evaluate the phospholipase activity and it was defined as follows [16]: Pz = 1, negative phospholipase activity; Pz = 0.90–0.99, very low phospholipase activity; Pz = 0.80–0.89, low phospholipase activity; Pz = 0.70–0.79, high phospholipase activity; Pz ≤ 0.69, very high phospholipase activity. All experiments were performed three times on different days.

### 2.9 Real-time quantitative PCR

CA10 cells at a final concentration of 2.5× 10^5^ CFU/mL were incubated in RPMI 1640 medium overnight at 35°C with the treatment using drugs alone or in combination at the following final concentrations: FLC at 1 μg/mL; BUD at 128 μg/mL. A sample without drug treatment was included as a control. After incubation, the cells were harvested for RNA extraction. The total RNA was extracted using an RNApure Yeast Kit (CWBiotech, Beijing, China). The first strand of cDNA was synthesized by reverse transcription using a First-Stand cDNA SynthesisSuperMix Kit (CWBiotech, Beijing, China). The thermal cycling condition was 95°C for 10 min as an initial denaturation step, followed by 40 cycles of 95°C for 15 s, 60°C for 1 min and 72°C for 32 s. The *ACT1* gene was used as an endogenous reference control for normalization of the relative expression. Drug transporter encoding genes (*CDR1*, *CDR2*, *MDR1* and *FLU1*), biofilm-related genes (*EFG1*, *HWP1* and *ALS1*), apoptosis-related genes (*MCA1*, *HSP90*, *CRZ1*, *CNA1*), and virulence factor-related genes (*PLB1*, *PLB2*, *PLB3*, *PLB4*, *PLB5*, *PLC1*) were determined by the realtime-PCR (RT-PCR) assay as mentioned above. Sequences of the primers are listed in Table 1. The result was calculated using the 2^-(ΔΔCt)^ method [26]. Graph Pad Prisma 5 software was used to analyze the results. All experiments were performed three times on different days.

**Table 1:** Primers used in this study.

Genes	Primer sequences(5’→3’)
*ACT1*	F:AGCCCAATCCAAAAGAGGTATT
	R:GCTTCGGTCAACAAAACTGG
*CDR1*	F:CAACACCAGGGAAACTTATCG
	R:TCTCGCAACACCATACCTCA
*CDR2*	F: TATTTCCGAGGTGGAGCACT
	R:GCAGATGGACGATAAAGAGCA
*MDR1*	F:GGTTTTCAGTCCGATGTCAG
	R:TAGCAAAGAATCCACCCAAA
*FLU1*	F: CGAGAAGAGCCACCAGAAGT
	R: GGACCATCAAAGGCAACAAC
*EFG1*	F:CCAACAGCAACAACAAAAGC
	R:GGGTGAAGGGTGAACTGAAC
*HWP1*	F: CAGCCACTGAAACACCAACT
	R: CAGAAGTAACAACAACAACACCAG
*ALS1*	F: TTGGGTTGGTCCTTAGATGG
	R: CACCATCGGCAGTTAAATCA
*MAC1*	F: AGATGCTCGTCCTAATGATGC
	R: TTGAGGCAATGTTTTCACCA
*HSP90*	F: GCTCCATTTGATGCCTTTGA
	R: CGACAACCCCCTTGATGA
*CRZ1*	F: CATCACAACCTGCATCTCCA
	R: GGTCAAGGTTCTGTGATCCAA
*CNA1*	F: TCATTACCGTTTGTGGGTGA
	R: TTGGGGTCACACCTATTTCAG
*PLB1*	F: GGGAACTCACACACTCATTTCTT
	R: AAGCATCACCTTCGTCTTCTG
*PLB2*	F: TGATACGGAACGACAAGGAA
	R: AATGGCACAACCAACACAAG
*PLB3*	F: TCTCCAAGTGGTGGATATGCT
	R: ATCGGCTTCACGGATAAATG
*PLB4*	F: CTGGACAATTAGCCGCATTA
	R: TACCATCCAATTCCCTCCTG
*PLB5*	F: TGATAAGCCAAGCACAAGCA
	R CGAGCCTGAACCAAACAAGT
*PLC1*	F: CATCTGGTCTGCCTCCTAAAT
	R: GCGGCGATTCACTATCATTA

## 3. Results

### 3.1 BUD increased the susceptibility of resistant *C*. *albicans* to FLC

To assess the nature and intensity of the *in vitro* interactions between FLC and BUD against *C*. *albicans* planktonic cells, the fractional inhibitory concentration index (FICI) model and the percentage of growth difference (△*E*) model were used in analyzing the data obtained from the checkerboard broth microdilution assays. The FIC index was calculated according to the following formula [27]: FICI = MIC_A comb_ /MIC_A alone_+MIC_B comb_ /MIC_B alone._ The interpretation of the FICI was as follows: FICI ≤ 0.5, synergistic effect; FICI > 0.5 but ≤ 4, no interaction; FICI > 4, antagonistic effect. As for the △*E* model, interactions with<100% were considered weak, interactions with 100%-200% were considered moderate, and interactions with >200% were considered strong, as described previously [27]. Our results showed strong synergistic antifungal interactions between FLC and BUD against resistant *C*. *albicans* strains *in vitro* (Table 2, S1 Table). No synergism was obtained with susceptible *C*. *albicans* strains (S1 Table). As shown in Table 2 and S1 Table, CA10 and CA16 became susceptible when the MICs of FLC were redetermined in the presence of BUD.

**Table 2:** *In vitro* drug effects evaluated by the FICI model and the △*E* model.

Drugs	Strains	MIC(μg/mL)				LA theory		BI theory		
		MIC_A_	C_A_	MIC_B_	C_B_	FICI	IN	∑SYN	∑ANT	IN
FLC+BUD	CA4	1	0.5	16	4	0.750	NI	24.63%	-199.56%	ANT
	CA8	2	1	32	16	1.000	NI	21.71%	-165.00%	ANT
	CA10	>512	1	>128	32	0.252	SYN	1822.28%	0	SYN
	CA16	>512	1	>128	32	0.252	SYN	1873.52%	0	SYN

MIC_80_ of each drug alone or in combination against *C*. *albicans* are shown as the median of three independent experiments. MIC_A_, The MICs of triazole antifungal agents when used alone; MIC_B_, The MICs of BUD when used alone; C_A_, The MICs of triazole antifungal agents when used in combination with BUD; C_B_, The MICs of BUD when used in combination with triazole antifungal agents; IN, interpretation; ANT, antagonism; SYN, synergism; NI, no interaction.

### 3.2 BUD combined with FLC had a synergistic effect against resistant *C*. *albicans in vivo*

#### Survival assay

**Fig 1:**
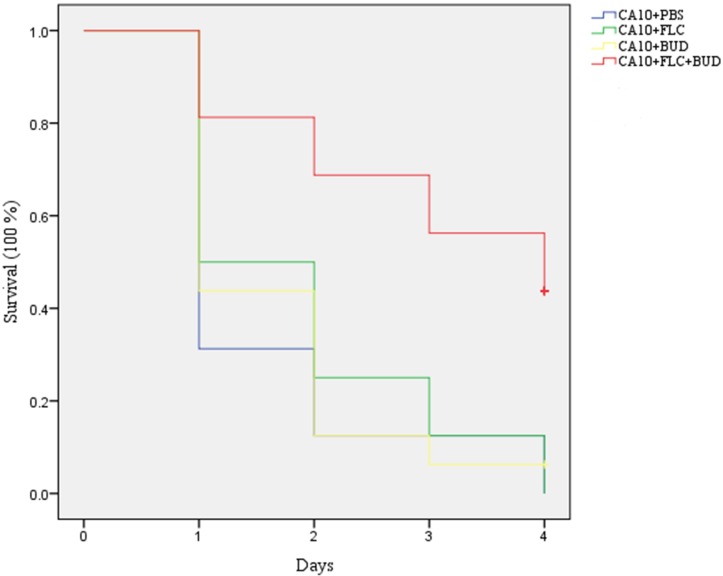
Efficacy of FLC alone or in combination with BUD on the survival of infected *G*. *mellonella* over 4 days. The concentration of yeast cells was 5×10^6^ CFU/larva. Treatments consisted of PBS, FLC (160μg/mL) alone, BUD (160μg/mL) alone, or a combination of FLC (160μg/mL) with BUD (160μg/mL).

In the present study, we compared the *G*. *mellonella* larvae survival rates of four groups to primarily evaluate the effects of the drug on fungal infected *G*. *mellonella* larvae. The survival rate for *G*. *mellonella* larvae treated with FLC or BUD alone was only a little higher than that of *G*. *mellonella* larvae treated with PBS (Fig 1, S2 Table). Notably, the survival rate of *G*. *mellonella* larvae was significantly higher when treated with the drugs in combination than that of *G*. *mellonella* larvae treated with PBS or the drug alone (*P* < 0.05), indicating that FLC and BUD have a synergistic antifungal effect on resistant *C*. *albicans in vivo* (Fig 1, S2 Table).

#### Fungal burden determination

**Fig 2:**
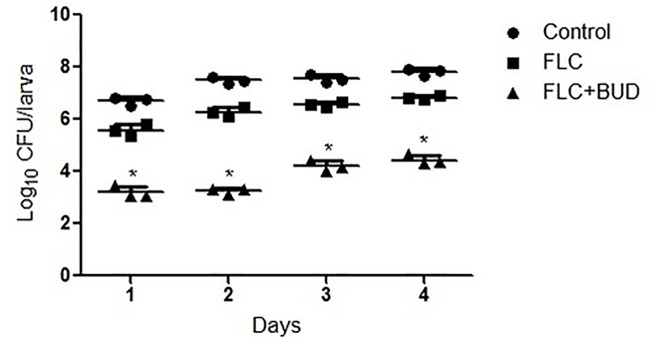
Fungal burden in infected *G*. *mellonella* over 4 days. The concentration of yeast cells was 5 × 10^6^ CFU/larva. Treatments consisted of PBS, FLC (160μg/mL) alone, BUD (160μg/mL) alone, or a combination of FLC (160μg/mL) with BUD (160μg/mL). Data came from the means of three independent experiments. For clarity, data for treatment with BUD are not shown because the data obtained closely followed those shown for the control group.

Fungal burden determination was performed to evaluate the drug’s effects on the tissue burden of *C*. *albicans* in *G*. *mellonella* larvae. Data showed that treatment with either FLC or BUD alone had almost no inhibitory effect, as there was a rapid proliferation of *C*. *albicans* within the *G*. *mellonella* larvae in these two groups over 4 days (Fig 2, S3 Table) and the recorded numbers of *C*. *albicans* in these two groups were similar to the control *G*. *mellonella* larvae. In contrast, reduction of the fungal burden was very significant in *G*. *mellonella* larvae treated with the combination of FLC and BUD (Fig 2, S3 Table).

#### Histology of larval tissue

**Fig 3:**
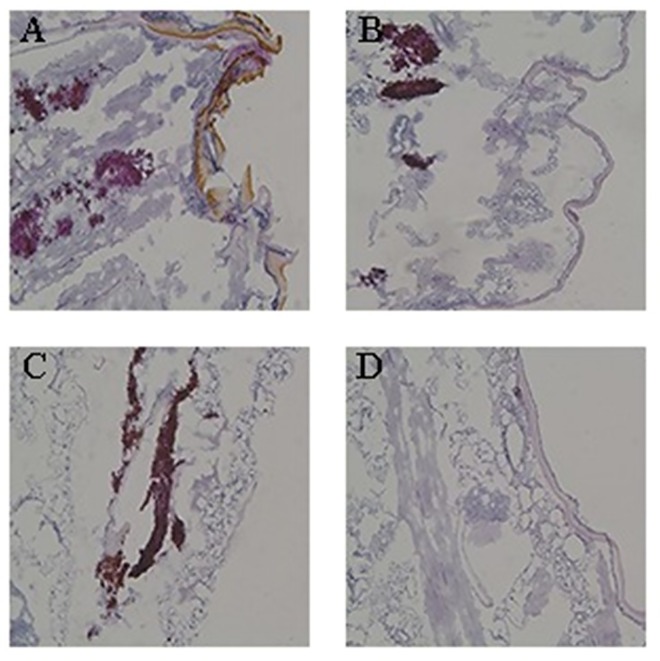
Histopathology of infected *G*. *mellonella* at day 2 past infection. The concentration of yeast cells was 5 × 10^6^ CFU/larva. Treatments consisted of PBS (A), 160μg/mL FLCalone (B), 160μg/mL BUD alone(C), or a combination of FLC and BUD (D). Melanized nodules was the mixtures of yeast cells and filaments.

To characterize the development of the fungal infection in *G*. *mellonella* larvae with the treatment of drugs, a histological study was performed by observing tissue sections after 2 days of infection. Drug combinations had a significant impact on the histology of infected *G*. *mellonella* larvae. The number of melanized nodules containing yeast cells and filaments in the larvae treated with FLC or BUD alone was almost the same as in the control *G*. *mellonella* larvae (Fig 3). Compared to untreated *G*. *mellonella* larvae or single drug treated *G*. *mellonella* larvae, a significant decrease in the number of melanized nodules was observed in *G*. *mellonella* larvae treated with a combination of FLC and BUD (Fig 3).

### 3.3 BUD increased the intracellular concentration of Rh6G by inhibiting drug transporter genes

**Fig 4:**
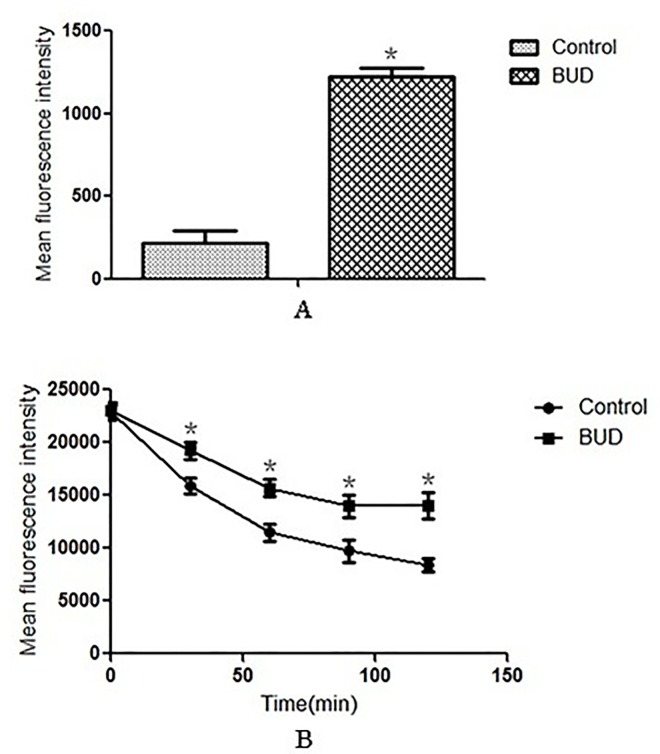
**The effect of BUD on the (A) uptake and (B) efflux of Rh6G in resistant *C*. *albicans*.** The uptake and efflux of Rh6G in the absence and presence of BUD (128μg/mL) was determined by a flow cytometry. MFIs represented the intracellular Rh6G in *C*. *albicans*. Error bars indicated standard errors of the means. Statistical significances were determined by Student’s t-test. *P < 0.05 when compared with the respective controls.

**Fig 5:**
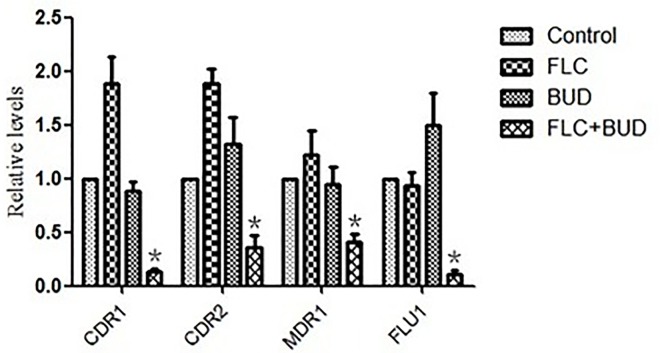
Relative expressions of *CDR1*, *CDR2*, *MDR1* and *FLU1* in resistant *C*. *albicans*. CA10 cells were treated with t no agent, 1μg/mL FLC, 128μg/mL BUD, and a combination of FLC and BUD at the same concentration. Error bars indicated standard errors of the means. Statistical significances were determined by Student’s t-test. *P < 0.05 when compared with the respective controls.

Both FLC and Rh6G are the substrates of drug transporters in *C*. *albicans*. To investigate the effect of BUD on FLC uptake and efflux, we assayed the uptake and efflux of Rh6G in *C*. *albicans* by flow cytometry and determined the expression changes of the drug transporter encoding genes *CDR1*, *CDR2*, *MDR1* and *FLU1* by RT-PCR. For Rh6G uptake, treatment with BUD induced a notable increase of MFI compared with the control group (Fig 4A, S4 Table), indicating that BUD can promote the uptake of extracellular FLC. For Rh6G efflux, the MFI both in the BUD group and the control group gradually decreased with the increase in experimental time, but the MFI in the BUD group was significantly lower than that of the control group (Fig 4B, S5 Table), indicating that BUD can inhibit the efflux of intracellular FLC. Interestingly, synergistic inhibitory effects on the expression of *CDR1*, *CDR2*, *MDR1* and *FLU1* were observed in cells treated with a combination of FLC and BUD (Fig 5, S9 Table). Of note, both FLC and BUD alone failed to inhibit the expression levels of these four genes (Fig 5, S9 Table). Above all, the BUD-induced synergistic effect on enhancing the susceptibility of resistant *C*. *albicans* to FLC may be due to BUD-induced uptake of FLC into cells and inhibition of the efflux of intracellular FLC.

### 3.4 BUD combined with FLC had synergistic effects against biofilms at different stages

**Fig 6:**
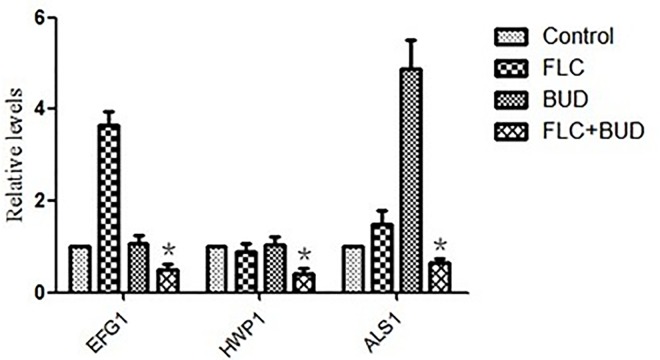
Relative expressions of *EFG1*, *HWP1* and *ALS1* in resistant *C*. *albicans*. CA10 cells were treated with t no agent, 1μg/mL FLC, 128μg/mL BUD, and a combination of FLC and BUD at the same concentration. Error bars indicated standard errors of the means. Statistical significances were determined by Student’s t-test. *P < 0.05 when compared with the respective controls.

The antifungal effects of drugs on *C*. *albicans* biofilms were evaluated using the FICI model as mentioned above. As shown in Table 3 and S6 Table, BUD combined with FLC showed synergistic antifungal effects against 4, 8, 12 and 24 h biofilms with FICI < 0.5. In addition, the synergism for the 12 and 24 h biofilms (FICI = 0.252, 0.258) was weaker than that of the 4 and 8 h biofilms (FICI = 0.127). *EFG1*, *HWP1*, and *ALS1* were closely involved in biofilm formation and development. Our data revealed that the expression levels of the biofilm-related genes *EFG1*, *HWP1*, and *ALS1* in *C*. *albicans* were significantly down-regulated with the combination treatment of FLC and BUD (*P* < 0.05), as shown in Fig 6 and S9 Table.

**Table 3:** Synergic effects of FLC alone and in combination with BUD against biofilms of resistant *C*. *albicans*.

Time(h)	SMIC(μg/mL)				LA theory	
	MIC_A_	C_A_	MIC_B_	C_B_	FICI	IN
4	>1024	2	128	16	0.127	SYN
8	>1024	2	128	16	0.127	SYN
12	>1024	2	512	128	0.252	SYN
24	>1024	8	>512	128	0.258	SYN

SMIC_80_ of each drug alone or in combination against *C*. *albicans* were shown as the median of three independent experiments. MIC_A_, The MICs of FLC when used alone; MIC_B_, The MICs of BUD when used alone; C_A_, The MICs of FLC when used in combination with BUD; C_B_, The MICs of BUD when used in combination with FLC; IN, interpretation; SYN, synergism.

### 3.5 BUD combined with FLC induced apoptosis

**Fig 7:**
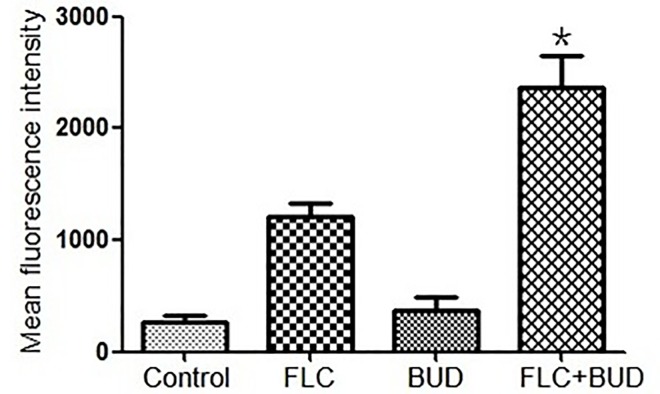
Changes of ROS in resistant *C*. *albicans*. CA10 cells with DHR-123 staining wrer analysed by the flow cytometry after treatments with no agent, 1μg/mL FLC, 128μg/mL BUD, or a combination of FLC and BUD at the same concentration. Error bars indicated standard errors of the means. Statistical significances were determined by Student’s t-test. *P < 0.05 when compared with the respective controls.

**Fig 8:**
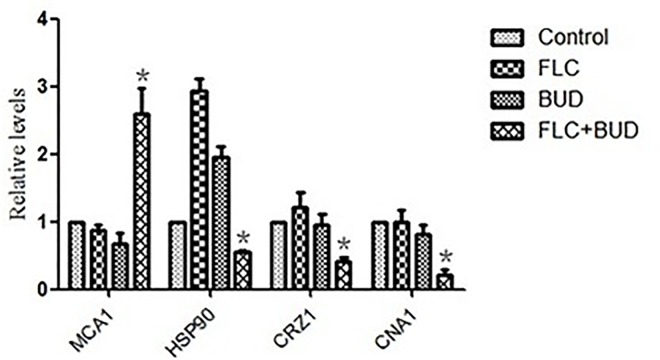
Relative expressions of *MAC1*, *HSP90*, *CRZ1* and *CNA1* in resistant *C*. *albicans*. CA10 cells were treated with t no agent, 1μg/mL FLC, 128μg/mL BUD, and a combination of FLC and BUD at the same concentration. Error bars indicated standard errors of the means. Statistical significances were determined by Student’s t-test. *P < 0.05 when compared with the respective controls.

Intracellular ROS in *C*. *albicans* was promoted, which was revealed by the significant fluorescence increases of DHR123 in the drug combination treatment group (Fig 7, S7 Table). The intensity of DHR123 fluorescence in the drug combination group increased by 9-, 2- and 6-fold compared with the control group, the FLC group and the BUD group, respectively (Fig 7, S7 Table). The up-regulation of *MAC1* induced by the combination treatment revealed that BUD combined with FLC can promote apoptosis in *C*. *albicans* (Fig 8, S9 Table). Other apoptosis-related genes, including *HSP90*, *CRZ1* and *CNA1*, were also reported to be involved in resistance, and their down-regulation demonstrated that BUD combined with FLC revises the resistance of *C*. *albicans* partly via inducing cellular apoptosis.

### 3.6 BUD combined with FLC inhibited the activity of extracellular phospholipase

**Fig 9:**
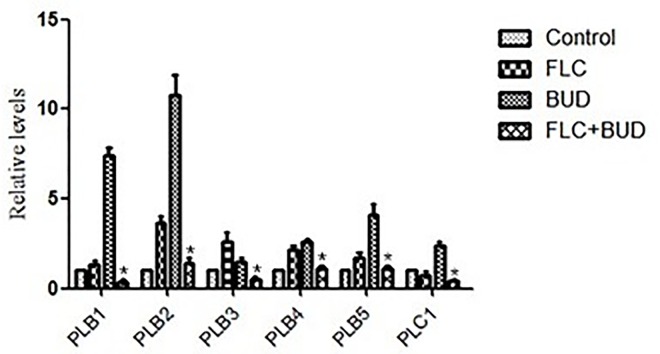
Relative expressions of *PLB1*-*5* and *PLC1* in resistant *C*. *albicans*. CA10 cells were treated with t no agent, 1μg/mL FLC, 128μg/mL BUD, and a combination of FLC and BUD at the same concentration. Error bars indicated standard errors of the means. Statistical significances were determined by Student’s t-test. *P < 0.05 when compared with the respective controls.

Pz values were obtained by averaging the triplicates. There was almost no difference in the Pz values between drug alone groups (0.64 ± 0.02 for FLC and 0.62 ± 0.02 for BUD) and the control group (0.62 ± 0.02), as shown in Table 4. Notably, no precipitation zone was observed in the drug combination group (Table 4, S8 Table), indicating that treatment with BUD combined with FLC could completely inhibit the extracellular phospholipase activity of resistant *C*. *albicans*. As shown in Fig 9 and S9 Table, phospholipase-related genes *PLB1*-*5* and *PLC1* in *C*. *albicans* were significantly down-regulated by the combination treatment of BUD and FLC (*P* < 0.05). These results told us that BUD combined with FLC resulted in synergistic antifungal effects involved in inhibiting the activity of extracellular phospholipase.

**Table 4:** Extracellular phospholipase activity of *C*. *albicans*.

Groups	Pz ^a^ values	Phospholipase activity
Control	0.62±0.01	Very high
FLC	0.64±0.02	Very high
BUD	0.62±0.02	Very high
FLC+BUD	NZ ^b^	Negative

^a^ *P* < 0.05 compared to the control.

^b^ NZ, no zone of precipitation.

## 4. Discussion

Our study showed that using such a combination of FLC and BUD can provide synergism against resistant *C*. *albicans in vitro*. No synergism has been obtained with susceptible *C*. *albicans in vitro*. As shown in Table 2 and S1 Table, CA10 and CA16 became susceptible when the MICs of FLC were redetermined in the presence of BUD using a checkerboard broth microdilution method. Both FICI and △*E* models revealed the synergistic effects of BUD and FLC against resistant *C*. *albicans in vitro*. These findings may also have implications for the prevention of drug resistance.

Insect infection models have significant ethical and economic advantages over mammalian infection models and can provide a rapid evaluation of the efficacy and toxicity of agents *in vivo*. *G*. *mellonella*, an insect infection model, has recently been used as an alternative to vertebrates for studying a number of important human pathogens [28]. The doses of FLC and BUD were chosen to mimic those used to treat human infections. Notably, exposure of infected *G*. *mellonella* larvae to a combination of FLC and BUD resulted in significantly enhanced survival (*P*<0.05) compared to monotherapy with either FLC or BUD (Fig 1, S2 Table). The fungal burden observed in the *G*. *mellonella* larvae correlated with the survival experiment. The measurement of *G*. *mellonella* larval burden in *C*. *albicans* showed that treatment with a combination of FLC and BUD had a synergistic inhibitory effect, as there was slower proliferation of *C*. *albicans* within the *G*. *mellonella* larvae over 4 days (Fig 2, S3 Table) and the numbers of *C*. *albicans* were decreased compared to those from infected *G*. *mellonella* larvae treated with PBS or drug alone (*P* < 0.05). To complement the studies *in vivo*, we further performed histopathology of infected *G*. *mellonella* larvae. Treatment with the drug alone hardly has any effect on the histopathology of the *G*. *mellonella* larvae, although the treatment with FLC and BUD simultaneously resulted in much fewer melanized nodules (Fig 3). Therefore, our data *in vivo* were in agreement with the antifungal effect determination *in vitro*. The mechanism involved in protecting *G*. *mellonella* larvae from *C*. *albicans* infection is unknown but might result from the immunomodulatory activities of BUD. It has been previously demonstrated that the highly frequent FLC resistance in *C*. *albicans* was in part attributed to the high expression of multi-drug transporters, which can result in decreased drug concentrations in the fungal cells [29, 30]. Two groups of drug transporters are found in *C*. *albicans*: the ATP-binding cassette (ABC) transporters and the major facilitator superfamily (MFS) transporters [31]. The products of *CDR1* and *CDR2* are ABC transporters, and the products of *MDR1* and *FLU1* are MFS-type transporters [32]. In our study, we found that there were significant inhibitory effects on the uptake and efflux of FLC in *C*. *albicans* treated with BUD (Fig 4, S4 and S5 Tables). In addition, we found that there was a significant down-regulation of drug transporter encoding genes *CDR1*, *CDR2*, *MDR1* and *FLU1* in the presence of BUD (Fig 5, S9 Table), suggesting that the BUD-mediated increased susceptibility of resistant *C*. *albicans* to FLC is at least in part through a mechanism of suppression of the expression of these drug transporters.

Accumulating evidence reveals that the formation of *C*. *albicans* biofilms could enhance the resistance of this pathogen to most of the commonly used antifungal agents [33–35]. High drug resistance conferred by *C*. *albicans* biofilms is recognized as a significant and growing clinical problem. Here, we found that BUD combined with FLC has significant synergistic effects against the resistant *C*. *albicans* biofilms in different stages (Table 3, S6 Table). Over the past years, the genetic network controlling the biofilm-associated antifungal drug resistance has been investigated and partially elucidated [36]. Among several groups of genes involved in biofilm formation, it was found that *EFG1*, *HWP1* and *ALS1* are remarkable [36–38]. *EFG1*, one of the transcription factors, is required for biofilm formation and has a certain role in the resistance of *C*. *albicans* biofilms to antifungals *in vivo* [39, 40]. *HWP1*, encoding a fungal cell wall protein, is required for hyphal development and yeast adhesion to epithelial cells as the initial step of biofilm colonization [38]. *ALS1*, encoding cell surface glycoproteins, exhibits high expression in *C*. *albicans* biofilm cells and also plays a key role in biofilm formation [36]. Significant decreases were found in the expressions of *EFG1*, *HWP1* and *ALS1* (Fig 6, S9 Table), which was in accordance with the anti-biofilm effects of FLC and BUD.

Recent studies have revealed that apoptosis may be a novel antifungal mechanism in *C*. *albicans* [41, 42]. Several studies have reported that ROS is both necessary and sufficient for inducing apoptosis in *C*. *albicans* [41, 43]. Increases in ROS are regarded as one of the typical hallmarks of early apoptosis [44–46]. BUD used with FLC in the present study resulted in a significantly increased level of ROS (*P* < 0.05), as shown in Fig 7 and S7 Table. Apoptosis is a complex process involving multiple factors, including *MCA1*, *HSP90*, *CRZ1* and *CNA1* [47–49]. *MCA1* is a central regulator of apoptosis and is involved in releasing ROS in fungi [43]. Hsp90 is a conserved molecular chaperone involved in the rapid development of drug resistance in *C*. *albicans*. It has been proven that Hsp90 potentiates the evolution of azole resistance in *C*. *albicans* via a calcineurin-dependent way [50]. *CRZ1* and *CNA1* are important elements of the calcineurin pathway, and both are reported to modulate azole resistance in *C*. *albicans* [48, 49]. To gain further insight into the molecular mechanism underlying induced apoptosis, we evaluated the expression levels of genes closely related to fungal apoptosis: *MCA1*, *HSP90*, *CRZ1* and *CNA1* [47]. *MAC1* was up-regulated, but *HSP90*, *CRZ1* and *CNA1* were down-regulated after treatment with BUD combined with FLC (Fig 8, S9 Table), indicating that apoptosis and its involved multiple factors play an important role in the proposed synergistic combination. We speculate that *MCA1* may also be relevant to the clinical acquisition of fluconazole resistance in *C*. *albicans*.

Extracellular phospholipase is pivotal to the virulence of *C*. *albicans*, making it a possible target for the development of novel antifungals [51]. The pathogenicity of *C*. *albicans* is regulated by many virulence factors and by their interactions with the immune response to the host. These virulence factors are primary sources of drug resistance development and invasive infections because they are difficult or impossible to eradicate using conventional anticandidal agents [52]. Of the virulence factors studied in *C*. *albicans*, only extracellular phospholipase activity was predictive of mortality [53]. Thus, phospholipase is considered a major virulence factor in *C*. *albicans*. In the present report, we demonstrated that BUD had a potential to increase the susceptibility of resistant *C*. *albicans* to FLC by suppressing the activity of extracellular phospholipase (Table 4, S8 Table) and inhibiting the expression of phospholipase encoding genes (Fig 9, S9 Table). These findings indicate that the inhibition of phospholipase activity could be a key mechanism of action by which BUD sensitizes the resistant *C*. *albicans* to FLC.

There have been few reports of iatrogenic Cushing syndrome caused by interactions between BUD and strong inhibitors of cytochrome CYP 3A4, mainly itraconazole [54, 55]. Inhaled BUD is a potent glucocorticoid that undergoes 99% first-pass metabolism through hepatic CYP 3A4. Although the oral adverse effects associated with inhaled corticosteroid use should not be ignored, the adverse effects of BUD are fairly low, which may due to its low systemic availability [56]. Despite the possibility that the strong CYP 3A4 inhibitor itraconazole in combination with BUD can cause complications, the inhibition of FLC on CYP 3A4 is much weaker than itraconazole and may subsequently cause fewer effects when combined with BUD in humans. In addition, the mechanisms and targets described in our study may help researchers design new FLC sensitizers with fewer side effects in the future, with the help of modern computer aids and structure-based selection techniques.

There have been few reports of iatrogenic Cushing syndrome caused by interaction between BUD and strong inhibitors of cytochrome CYP 3A4 mainly itraconazole [54, 55]. Inhaled BUD is a potent glucocorticoid that undergoes 99% first-pass metabolism through hepatic CYP 3A4. Although the oral adverse effects associated with inhaled corticosteroid use should not be ignored, the adverse effects of BUD are fairly low, which may due to its low systemic availability [56]. Despite the strong CYP 3A4 inhibitor itraconazole in combination with BUD can cause complications, the inhibition of FLC on CYP 3A4 is much weaker than itraconazole and may cause few subsequent effects of BUD in human. In addition, the mechanisms and targets described in our study may help researchers designing new FLC sensitizers with few side effects in the future, with the help of modern computer aid and structure-based selecting techniques.

## 5. Conclusion

In conclusion, we first found that BUD can work synergistically with FLC against resistant *C*. *albicans* both *in vitro* and *in vivo*. These findings indicated that glucocorticoids enhance the anti-infection effect through not only anti-inflammatory effects but also a combined antifungal effect. Although the *G*. *mellonella* infection model has been used to test the efficacy of antifungals, this model still has limitations. The efficacy of FLC combined with BUD *in vivo* should be further evaluated using a mammalian model in future studies. Mechanism research demonstrated that this synergism is related to the inhibition of drug transporter function, the reduction of the formation of biofilms, the promotion of apoptosis and the inhibition of the activity of extracellular phospholipases. RT-PCR results revealed many potential targets for competing drug resistance in *C*. *albicans*, namely drug transporter genes (*CDR1*, *CDR2*, *MDR1* and *FLU1*), biofilm-related genes (*EFG1*, *HWP1* and *ALS1*), apoptosis-related genes (*MAC1*, *HSP90*, *CRZ1* and *CNA1*) and phospholipases encoding genes (*PLB1-5* and *PLC1*). This is the first study outlining the mechanisms of BUD against *C*. *albicans* alone or in combination with FLC. In addition, our study may lead to the identification of new potential antifungal targets and promote research on the *in vitro* effects of other glucocorticoids on *C*. *albicans*.

## Supporting Information

S1 Table The data for *in vitro* drug effects against four *C*. *albicans* strains. The growth rates of *C*. *albicans* CA4, CA8, CA10 and CA16 after treatment with FLC alone or in combination with BUD are shown in S1a, 1b, 1c and 1d Table, respectively.
(DOC)

S2 Table The data for the efficacy of FLC alone or in combination with BUD on the survival of infected *G*. *mellonella* over 4 days. (DOC)

S3 Table The data for fungal burden in infected *G*. *mellonella* over 4 days. (DOC)

S4 Table The data for the effect of BUD on the uptake of Rh6G in resistant *C*. *albicans*. (DOC)

S5 Table The data for the effect of BUD on the efflux of Rh6G in resistant *C*. *albicans*. (DOC)

S6 Table The data for the synergistic effects of FLC alone and in combination with BUD against biofilms of resistant *C*. *albicans*. The growth rates of *C*. *albicans* biofilms at 4, 8, 12 and 24 h after treatment with FLC alone or in combination with BUD are shown in S6a, 6b, 6c and 6d Table, respectively.
(DOC)

S7 Table The data for changes in ROS in resistant *C*. *albicans*. (DOC)

S8 Table The data for extracellular phospholipase activity in resistant *C*. *albicans*. (DOC)

S9 Table The data for the relative expressions of genes in resistant *C*. *albicans*. (DOC)
