# Supplementary material for: Synergistic Effects and Mechanisms of Budesonide in Combination with Fluconazole against Resistant Candida albicans
Source: PLoS One. 2016 Dec 22;11(12):e0168936. doi: 10.1371/journal.pone.0168936 (PMC5179115; doi:10.1371/journal.pone.0168936)
Supplement: S1 Table — The growth rates of C. albicans CA4, CA8, CA10 and CA16 after treatment with FLC alone or in combination with BUD are shown in S1a, 1b, 1c and 1d Table, respectively. (DOC) [file pone.0168936.s001.doc]

S1a Table. The data for *in vitro* drug effects against susceptible *C. albicans* CA4

|  | | **FLC (μg/ml)** | | | | | | | | | | | |
| --- | --- | --- | --- | --- | --- | --- | --- | --- | --- | --- | --- | --- | --- |
|  |  | **0** | **0.002** | **0.004** | **0.008** | **0.016** | **0.032** | **0.063** | **0.125** | **0.25** | **0.5** | **1** | **Blank** |
| **BUD (μg/ml)** | **128** | 18.0% | 16.21% | 15.32% | 17.17% | 13.61% | 12.49% | 10.11% | 10.71% | 9.14% | 9.00% | 8.62% | 0 |
|  | **64** | 18.81% | 18.59% | 18.51% | 17.77% | 16.80% | 15.76% | 14.94% | 14.57% | 13.90% | 13.46% | 9.89% | 0 |
|  | **32** | 19.03% | 18.74% | 18.51% | 18.44% | 17.77% | 17.47% | 16.95% | 16.73% | 16.43% | 16.06% | 11.45% | 0 |
|  | **16** | 19.85% | 19.03% | 19.11% | 18.59% | 18.44% | 17.55% | 17.03% | 16.80% | 16.58% | 16.21% | 10.04% | 0 |
|  | **8** | 96.21% | 90.56% | 92.91% | 93.23% | 95.24% | 92.71% | 92.90% | 92.04% | 30.11% | 17.62% | 9.44% | 0 |
|  | **4** | 98.68% | 99.33% | 99.63% | 99.93% | 95.76% | 95.61% | 95.32% | 93.68% | 31.67% | 18.59% | 11.45% | 0 |
|  | **2** | 99.33% | 99.85% | 99.33% | 96.51% | 96.51% | 98.14% | 97.77% | 94.65% | 32.86% | 22.23% | 11.67% | 0 |
|  | **0** | 100.00% | 99.93% | 99.41% | 98.74% | 98.44% | 97.77% | 97.84% | 96.88% | 33.09% | 29.14% | 11.82% | 0 |

S1b Table. The data for *in vitro* drug effects against susceptible *C. albicans* CA8

|  | | **FLC (μg/ml)** | | | | | | | | | | | |
| --- | --- | --- | --- | --- | --- | --- | --- | --- | --- | --- | --- | --- | --- |
|  |  | **0** | **0.004** | **0.008** | **0.016** | **0.032** | **0.063** | **0.125** | **0.25** | **0.5** | **1** | **2** | **Blank** |
| **BUD (μg/ml)** | **128** | 17.63% | 17.85% | 17.92% | 17.63% | 16.96% | 16.67% | 16.37% | 14.97% | 14.01% | 12.76% | 6.49% | 0 |
|  | **64** | 17.48% | 19.17% | 18.51% | 18.44% | 18.07% | 16.96% | 17.04% | 15.19% | 14.68% | 16.00% | 9.00% | 0 |
|  | **32** | 19.69% | 19.62% | 19.40% | 18.95% | 19.54% | 19.03% | 18.88% | 17.55% | 17.85% | 17.33% | 8.48% | 0 |
|  | **16** | 38.04% | 36.36% | 36.06% | 35.25% | 34.88% | 34.00% | 32.37% | 31.71% | 30.53% | 18.58% | 8.55% | 0 |
|  | **8** | 93.58% | 92.70% | 91.08% | 92.55% | 91.45% | 88.86% | 88.57% | 79.65% | 63.94% | 21.31% | 9.07% | 0 |
|  | **4** | 98.82% | 99.04% | 98.75% | 97.86% | 97.49% | 96.46% | 95.94% | 80.60% | 74.48% | 29.94% | 9.51% | 0 |
|  | **2** | 99.78% | 99.41% | 98.60% | 98.30% | 97.71% | 96.98% | 96.39% | 95.87% | 75.29% | 29.28% | 10.03% | 0 |
|  | **0** | 100.00% | 99.63% | 98.97% | 98.75% | 98.67% | 97.71% | 96.98% | 96.31% | 76.55% | 29.87% | 11.28% | 0 |

S1c Table. The data for *in vitro* drug effects against resistant *C. albicans* CA10

|  | | **FLC (μg/ml)** | | | | | | | | | | | |
| --- | --- | --- | --- | --- | --- | --- | --- | --- | --- | --- | --- | --- | --- |
|  |  | **0** | **0.125** | **0.25** | **0.5** | **1** | **2** | **4** | **8** | **16** | **32** | **64** | **Blank** |
| **BUD (μg/ml)** | **128** | 46.69% | 0.68% | 0.53% | 0.35% | 0.33% | 0.60% | 0.69% | 0.83% | 0.42% | 0.35% | 1.87% | 0 |
|  | **64** | 98.12% | 60.49% | 10.04% | 3.67% | 3.89% | 0.41% | 0.37% | 0.32% | 0.07% | 1.01% | 1.01% | 0 |
|  | **32** | 98.28% | 66.42% | 37.17% | 22.61% | 13.61% | 5.92% | 0.63% | 1.40% | 3.24% | 2.01% | 0.14% | 0 |
|  | **16** | 98.27% | 69.20% | 59.54% | 51.99% | 47.21% | 49.36% | 27.22% | 25.70% | 23.34% | 22.39% | 20.54% | 0 |
|  | **8** | 98.97% | 72.00% | 66.60% | 60.42% | 55.46% | 61.74% | 45.68% | 43.98% | 43.95% | 41.40% | 34.67% | 0 |
|  | **4** | 99.01% | 73.80% | 72.40% | 61.37% | 59.46% | 61.66% | 51.17% | 59.33% | 55.89% | 46.65% | 46.93% | 0 |
|  | **2** | 99.19% | 73.62% | 73.24% | 64.75% | 65.67% | 63.08% | 58.40% | 58.99% | 57.11% | 52.76% | 50.28% | 0 |
|  | **0** | 100.00% | 77.54% | 75.60% | 70.12% | 67.16% | 65.78% | 65.65% | 63.49% | 60.97% | 57.61% | 52.65% | 0 |

S1d Table. The data for *in vitro* drug effects against resistant *C. albicans* CA16

|  | | **FLC (****μg/ml)** | | | | | | | | | | | |
| --- | --- | --- | --- | --- | --- | --- | --- | --- | --- | --- | --- | --- | --- |
|  |  | **0** | **0.125** | **0.25** | **0.5** | **1** | **2** | **4** | **8** | **16** | **32** | **64** | **Blank** |
| **BUD (μg/ml)** | **128** | 51.79% | 0.14% | 0.08% | 0.10% | 0.08% | 0.19% | 0.08% | 0.14% | 0.06% | 0.03% | 0.02% | 0 |
|  | **64** | 96.46% | 12.02% | 1.32% | 0.60% | 0.46% | 0.45% | 0.43% | 0.38% | 0.34% | 0.32% | 0.16% | 0 |
|  | **32** | 97.57% | 31.27% | 29.90% | 27.89% | 12.36% | 12.83% | 12.59% | 12.51% | 12.53% | 9.47% | 8.44% | 0 |
|  | **16** | 97.96% | 76.53% | 40.96% | 41.02% | 40.52% | 33.27% | 31.16% | 29.38% | 28.31% | 27.35% | 27.25% | 0 |
|  | **8** | 98.23% | 77.41% | 62.11% | 43.18% | 42.13% | 41.20% | 40.35% | 40.25% | 39.55% | 38.49% | 39.11% | 0 |
|  | **4** | 98.88% | 77.20% | 70.22% | 59.00% | 58.00% | 54.62% | 54.06% | 52.86% | 48.70% | 47.58% | 45.81% | 0 |
|  | **2** | 99.48% | 80.08% | 78.75% | 62.04% | 59.66% | 60.48% | 59.37% | 56.69% | 56.23% | 53.91% | 51.02% | 0 |
|  | **0** | 100.00% | 80.94% | 80.22% | 64.56% | 61.89% | 61.57% | 60.06% | 59.09% | 57.89% | 56.87% | 55.04% | 0 |

Abbreviation: FLC: fluconazole; BUD: budesonide.
