# Supplementary material for: Synergistic Effects and Mechanisms of Budesonide in Combination with Fluconazole against Resistant Candida albicans
Source: PLoS One. 2016 Dec 22;11(12):e0168936. doi: 10.1371/journal.pone.0168936 (PMC5179115; doi:10.1371/journal.pone.0168936)
Supplement: S2 Table — (DOC) [file pone.0168936.s002.doc]

S2 Table. The data for efficacy of FLC alone or in combination with BUD on the survival of infected *G. mellonella* over 4 days.

| Days | Survival larvae number (survival rate) | | | |
| --- | --- | --- | --- | --- |
|  | Control | FLC | BUD | FLC+BUD |
| 1 | 5(31.25%) | 8(50.00%) | 7(43.75%) | 13(81.25%) |
| 2 | 2(12.50%) | 4(25.00%) | 2(12.50%) | 11(68.75%) |
| 3 | 0 | 2(12.50%) | 1(6.25%) | 9(56.25%) |
| 4 | 0 | 0 | 1(6.25%) | 7(43.75%) |

Abbreviation: FLC: fluconazole; BUD: budesonide.
