# Supplementary material for: Synergistic Effects and Mechanisms of Budesonide in Combination with Fluconazole against Resistant Candida albicans
Source: PLoS One. 2016 Dec 22;11(12):e0168936. doi: 10.1371/journal.pone.0168936 (PMC5179115; doi:10.1371/journal.pone.0168936)
Supplement: S3 Table — (DOC) [file pone.0168936.s003.doc]

S3 Table. The data for fungal burden in infected *G. mellonella* over 4 days.

| Days | CFU/larva | | | | | | | | | | | |
| --- | --- | --- | --- | --- | --- | --- | --- | --- | --- | --- | --- | --- |
|  | Control | | | FLC | | | BUD | | | FLC+BUD | | |
| 1 | 5000000 | 5500000 | 2500000 | 400000 | 560000 | 330000 | 3000000 | 2500000 | 800000 | 1500 | 800 | 1000 |
| 1 | 8000000 | 4400000 | 2000000 | 200000 | 580000 | 300000 | 2000000 | 2700000 | 1600000 | 2500 | 1000 | 1500 |
| 1 | 3500000 | 10000000 | 5000000 | 100000 | 850000 | 400000 | 1000000 | 5500000 | 2000000 | 4500 | 1500 | 1000 |
| 2 | 36000000 | 42000000 | 40000000 | 2800000 | 2800000 | 1600000 | 2000000 | 2500000 | 700000 | 3000 | 2000 | 700 |
| 2 | 20000000 | 44000000 | 30000000 | 1100000 | 2200000 | 900000 | 4000000 | 8000000 | 7000000 | 2400 | 1200 | 2000 |
| 2 | 15000000 | 40000000 | 20000000 | 1800000 | 3500000 | 1400000 | 7000000 | 6000000 | 2500000 | 1000 | 3000 | 1000 |
| 3 | 30000000 | 50000000 | 20000000 | 2000000 | 4000000 | 500000 | 35000000 | 20000000 | 19000000 | 9000 | 25000 | 8000 |
| 3 | 10000000 | 37000000 | 18000000 | 5000000 | 5200000 | 3000000 | 10000000 | 28000000 | 10000000 | 15000 | 30000 | 9000 |
| 3 | 60000000 | 70000000 | 35000000 | 3900000 | 3800000 | 5000000 | 40000000 | 60000000 | 30000000 | 20000 | 22000 | 15000 |
| 4 | 50000000 | 35000000 | 80000000 | 6000000 | 7000000 | 6000000 | 35000000 | 60000000 | 50000000 | 10000 | 45000 | 10000 |
| 4 | 90000000 | 50000000 | 80000000 | 4500000 | 7300000 | 7000000 | 65000000 | 55000000 | 25000000 | 35000 | 55000 | 20000 |
| 4 | 70000000 | 60000000 | 90000000 | 8900000 | 9000000 | 5000000 | 70000000 | 70000000 | 60000000 | 20000 | 32000 | 30000 |

Abbreviation: FLC: fluconazole; BUD: budesonide.
