# Supplementary material for: Synergistic Effects and Mechanisms of Budesonide in Combination with Fluconazole against Resistant Candida albicans
Source: PLoS One. 2016 Dec 22;11(12):e0168936. doi: 10.1371/journal.pone.0168936 (PMC5179115; doi:10.1371/journal.pone.0168936)
Supplement: S4 Table — (DOC) [file pone.0168936.s004.doc]

S4 Table. The data for effect of BUD on the uptake of Rh6G in resistant *C. albicans*

| Groups | Mean fluorescence intensity |
| --- | --- |
| Control | 373 |
|  | 145 |
|  | 128 |
| BUD | 1327 |
|  | 1133 |
|  | 1203 |

Abbreviation: BUD: budesonide.
