# Supplementary material for: Synergistic Effects and Mechanisms of Budesonide in Combination with Fluconazole against Resistant Candida albicans
Source: PLoS One. 2016 Dec 22;11(12):e0168936. doi: 10.1371/journal.pone.0168936 (PMC5179115; doi:10.1371/journal.pone.0168936)
Supplement: S5 Table — (DOC) [file pone.0168936.s005.doc]

S5 Table. The data for effect of BUD on the efflux of Rh6G in resistant *C. albicans*.

| Groups | Mean fluorescence intensity | | | | |
| --- | --- | --- | --- | --- | --- |
|  | 0 min | 30 min | 60 min | 90 min | 120 min |
| Control | 22102 | 14941 | 10433. | 8107 | 7359. |
|  | 22185 | 15311. | 11002 | 9354 | 8536 |
|  | 24518 | 17411 | 12941. | 11741 | 9293 |
| BUD | 22726 | 18107 | 14503. | 12753 | 12644 |
|  | 22129 | 18753 | 15404 | 13130 | 12927 |
|  | 22129 | 20835 | 17204. | 16081 | 16506 |

Abbreviation: BUD: budesonide.
