# Supplementary material for: Synergistic Effects and Mechanisms of Budesonide in Combination with Fluconazole against Resistant Candida albicans
Source: PLoS One. 2016 Dec 22;11(12):e0168936. doi: 10.1371/journal.pone.0168936 (PMC5179115; doi:10.1371/journal.pone.0168936)
Supplement: S6 Table — The growth rates of C. albicans biofilms at 4, 8, 12 and 24 h after treatment with FLC alone or in combination with BUD are shown in S6a, 6b, 6c and 6d Table, respectively. (DOC) [file pone.0168936.s006.doc]

S6a Table. The data for [synergistic](javascript:void(0);) effects of FLC alone and in combination with BUD against 4h biofilm of resistant *C. albicans*

|  | | **FLC (μg/ml)** | | | | | | | | | | | |
| --- | --- | --- | --- | --- | --- | --- | --- | --- | --- | --- | --- | --- | --- |
|  |  | 0 | 2 | 4 | 8 | 16 | 32 | 64 | 128 | 256 | 512 | 1024 | Blank |
| **BUD (μg/ml)** | **128** | 7.61% | 5.18% | 2.21% | 0.65% | 0.76% | 0.01% | 0.09% | 0.65% | 0.11% | 0.08% | 0.05% | 0 |
|  | **64** | 36.40% | 30.14% | 9.44% | 8.44% | 4.63% | 2.00% | 1.59% | 0.97% | 0.32% | 0.77% | 0.07% | 0 |
|  | **32** | 59.90% | 14.46% | 12.73% | 12.62% | 12.06% | 11.36% | 11.26% | 10.60% | 7.24% | 3.85% | 2.69% | 0 |
|  | **16** | 77.83% | 19.73% | 19.11% | 18.77% | 17.48% | 16.77% | 14.42% | 13.26% | 12.50% | 10.76% | 10.55% | 0 |
|  | **8** | 78.49% | 61.43% | 51.76% | 49.75% | 47.85% | 47.67% | 46.25% | 45.83% | 44.76% | 43.59% | 41.52% | 0 |
|  | **4** | 80.78% | 79.80% | 78.41% | 78.44% | 76.02% | 75.58% | 73.69% | 74.71% | 74.17% | 73.73% | 68.80% | 0 |
|  | **2** | 80.93% | 80.14% | 78.86% | 78.56% | 77.15% | 77.74% | 76.82% | 75.45% | 75.65% | 75.35% | 71.94% | 0 |
|  | **0** | 100.00% | 98.76% | 89.33% | 88.34% | 89.79% | 89.88% | 89.60% | 78.57% | 76.67% | 75.49% | 73.57% | 0 |

S6b Table. The datas for [synergistic](javascript:void(0);) effects of FLC alone and in combination with BUD against 8h biofilm of resistant *C. albicans*

|  | | **FLC (μg/ml)** | | | | | | | | | | | |
| --- | --- | --- | --- | --- | --- | --- | --- | --- | --- | --- | --- | --- | --- |
|  |  | **0** | **2** | **4** | **8** | **16** | **32** | **64** | **128** | **256** | **512** | **1024** | Blank |
| **BUD (μg/ml)** | **128** | 10.58% | 9.71% | 9.68% | 1.03% | 0.28% | 0.31% | 0.23% | 0.20% | 0.17% | 0.10% | 0.06% | 0 |
|  | **64** | 27.65% | 33.61% | 10.28% | 9.48% | 9.60% | 9.67% | 6.39% | 5.46% | 4.25% | 2.63% | 0.41% | 0 |
|  | **32** | 42.42% | 73.81% | 70.36% | 46.32% | 42.80% | 38.04% | 37.57% | 31.62% | 28.51% | 26.31% | 25.45% | 0 |
|  | **16** | 61.22% | 19.72% | 19.19% | 18.15% | 17.95% | 17.12% | 16.71% | 15.57% | 13.69% | 12.15% | 10.76% | 0 |
|  | **8** | 79.27% | 51.42% | 49.08% | 48.67% | 48.25% | 47.65% | 45.05% | 43.64% | 42.38% | 41.92% | 39.62% | 0 |
|  | **4** | 80.75% | 73.61% | 71.35% | 67.61% | 66.48% | 65.68% | 64.71% | 63.71% | 61.20% | 60.31% | 54.22% | 0 |
|  | **2** | 90.77% | 85.86% | 83.18% | 82.07% | 80.39% | 80.33% | 81.70% | 76.20% | 73.85% | 70.55% | 67.15% | 0 |
|  | **0** | 100.00% | 96.92% | 89.59% | 88.10% | 87.95% | 86.62% | 86.06% | 76.80% | 74.23% | 71.30% | 68.46% | 0 |

S6c Table. The datas for [synergistic](javascript:void(0);) effects of FLC alone and in combination with BUD against 12h biofilm of resistant *C. albicans*

|  | | **FLC (μg/ml)** | | | | | | | | | | | |
| --- | --- | --- | --- | --- | --- | --- | --- | --- | --- | --- | --- | --- | --- |
|  |  | **0** | **2** | **4** | **8** | **16** | **32** | **64** | **128** | **256** | **512** | **1024** | **Blank** |
| **BUD (μg/ml)** | **512** | 17.34% | 1.33% | 1.26% | 1.19% | 0.47% | 0.03% | 0.15% | 0.51% | 0.19% | 0.15% | 0.29% | 0 |
|  | **256** | 27.63% | 2.46% | 1.57% | 2.22% | 1.50% | 1.47% | 1.31% | 1.14% | 1.01% | 0.83% | 0.50% | 0 |
|  | **128** | 45.33% | 18.44% | 17.44% | 16.11% | 15.98% | 15.62% | 15.59% | 13.32% | 12.50% | 11.71% | 9.56% | 0 |
|  | **64** | 65.49% | 48.03% | 47.91% | 48.08% | 46.65% | 44.48% | 42.97% | 40.06% | 38.12% | 35.76% | 33.38% | 0 |
|  | **32** | 87.00% | 78.98% | 75.12% | 71.81% | 66.59% | 64.91% | 65.08% | 63.57% | 59.97% | 59.83% | 58.90% | 0 |
|  | **16** | 98.15% | 79.69% | 79.76% | 77.59% | 75.57% | 75.28% | 74.44% | 74.78% | 74.70% | 71.63% | 67.99% | 0 |
|  | **8** | 98.45% | 81.91% | 80.45% | 76.71% | 85.96% | 84.03% | 84.78% | 76.95% | 76.95% | 73.23% | 72.34% | 0 |
|  | **0** | 100.00% | 99.90% | 90.73% | 88.24% | 88.02% | 85.30% | 84.14% | 81.29% | 77.52% | 74.55% | 73.05% | 0 |

S6d Table. The datas for [synergistic](javascript:void(0);) effects of FLC alone and in combination with BUD against 24h biofilm of resistant *C. albicans*

|  | | **FLC (****μg/ml)** | | | | | | | | | | | |
| --- | --- | --- | --- | --- | --- | --- | --- | --- | --- | --- | --- | --- | --- |
|  |  | **0** | **2** | **4** | **8** | **16** | **32** | **64** | **128** | **256** | **512** | **1024** | **Blank** |
| **BUD (μg/ml)** | **512** | 87.65% | 1.00% | 0.99% | 0.78% | 0.77% | 1.29% | 1.12% | 1.25% | 0.85% | 0.80% | 1.04% | 0 |
|  | **256** | 93.55% | 19.35% | 17.63% | 16.03% | 13.05% | 10.89% | 10.94% | 10.72% | 10.75% | 8.15% | 5.51% | 0 |
|  | **128** | 93.72% | 48.70% | 37.70% | 19.91% | 18.61% | 18.42% | 17.59% | 15.46% | 13.85% | 12.94% | 10.79% | 0 |
|  | **64** | 93.61% | 72.21% | 58.45% | 46.07% | 35.17% | 36.83% | 27.84% | 27.16% | 24.28% | 20.30% | 19.50% | 0 |
|  | **32** | 94.15% | 65.10% | 63.19% | 55.40% | 54.80% | 60.55% | 41.19% | 42.95% | 39.64% | 36.36% | 33.57% | 0 |
|  | **16** | 94.10% | 72.87% | 71.93% | 58.73% | 56.47% | 60.14% | 46.67% | 57.24% | 51.58% | 41.95% | 44.69% | 0 |
|  | **8** | 94.09% | 92.75% | 91.66% | 88.86% | 85.01% | 83.31% | 80.31% | 79.61% | 78.92% | 79.19% | 76.30% | 0 |
|  | **0** | 94.74% | 93.55% | 92.22% | 89.38% | 86.21% | 84.87% | 84.58% | 81.89% | 80.30% | 81.28% | 79.68% | 0 |

Abbreviation: FLC: fluconazole; BUD: budesonide.
