# Supplementary material for: Synergistic Effects and Mechanisms of Budesonide in Combination with Fluconazole against Resistant Candida albicans
Source: PLoS One. 2016 Dec 22;11(12):e0168936. doi: 10.1371/journal.pone.0168936 (PMC5179115; doi:10.1371/journal.pone.0168936)
Supplement: S7 Table — (DOC) [file pone.0168936.s007.doc]

S7 Table. The data for changes of ROS in resistant *C. albicans.*

| Groups | Mean fluorescence intensity |
| --- | --- |
| Control | 157 |
|  | 269 |
|  | 358 |
| FLC | 964 |
|  | 1242 |
|  | 1407 |
| BUD | 138 |
|  | 396 |
|  | 576 |
| FLC+BUD | 1938 |
|  | 2221 |
|  | 2917 |

Abbreviation: FLC: fluconazole; BUD: budesonide.
