# Supplementary material for: Synergistic Effects and Mechanisms of Budesonide in Combination with Fluconazole against Resistant Candida albicans
Source: PLoS One. 2016 Dec 22;11(12):e0168936. doi: 10.1371/journal.pone.0168936 (PMC5179115; doi:10.1371/journal.pone.0168936)
Supplement: S8 Table — (DOC) [file pone.0168936.s008.doc]

S8 Table. The data for extracellular phospholipase activity of resistant *C. albicans.*

| Groups | Pz values |
| --- | --- |
| Control | 0.608 |
|  | 0.636 |
|  | 0.6269 |
| FLC | 0.622 |
|  | 0.667 |
|  | 0.6458 |
| BUD | 0.604 |
|  | 0.642 |
|  | 0.625 |
| FLC+BUD | NZ |
|  | NZ |
|  | NZ |

Abbreviation: FLC: fluconazole; BUD: budesonide; NZ: no zone of precipitation.
