# Supplementary material for: Synergistic Effects and Mechanisms of Budesonide in Combination with Fluconazole against Resistant Candida albicans
Source: PLoS One. 2016 Dec 22;11(12):e0168936. doi: 10.1371/journal.pone.0168936 (PMC5179115; doi:10.1371/journal.pone.0168936)
Supplement: S9 Table — (DOC) [file pone.0168936.s009.doc]

S9 Table. The data for relative expressions of genes in resistant *C. albicans*.

| Genes | Relative expression levels | | | |
| --- | --- | --- | --- | --- |
|  | Control | FLC | BUD | FLC+BUD |
| *CDR1* | 1 | 2.316 | 0.711 | 0.126 |
|  | 1 | 1.466 | 0.979 | 0.188 |
|  | 1 | 1.882 | 0.951 | 0.104 |
| *CDR2* | 1 | 2.154 | 1.657 | 0.576 |
|  | 1 | 1.791 | 1.485 | 0.221 |
|  | 1 | 1.696 | 0.808 | 0.276 |
| *MDR1* | 1 | 1.563 | 0.688 | 0.514 |
|  | 1 | 0.819 | 0.925 | 0.442 |
|  | 1 | 1.287 | 1.243 | 0.265 |
| *FLU1* | 1 | 0.986 | 1.555 | 0.055 |
|  | 1 | 1.111 | 0.947 | 0.154 |
|  | 1 | 0.718 | 1.985 | 0.133 |
| *EFG1* | 1 | 3.735 | 1.424 | 0.693 |
|  | 1 | 4.123 | 0.807 | 0.334 |
|  | 1 | 3.123 | 1.011 | 0.507 |
| *HWP1* | 1 | 0.550 | 0.696 | 0.348 |
|  | 1 | 0.883 | 1.333 | 0.287 |
|  | 1 | 1.211 | 1.083 | 0.617 |
| *ALS1* | 1 | 1.012 | 3.640 | 0.438 |
|  | 1 | 1.491 | 5.732 | 0.819 |
|  | 1 | 1.983 | 5.285 | 0.674 |
| *MAC1* | 1 | 0.929 | 0.962 | 2.026 |
|  | 1 | 0.962 | 0.988 | 3.279 |
|  | 1 | 0.962 | 0.534 | 2.493 |
| *HSP90* | 1 | 2.980 | 1.685 | 0.593 |
|  | 1 | 3.230 | 1.913 | 0.484 |
|  | 1 | 2.560 | 2.245 | 0.566 |
| *CRZ1* | 1 | 1.563 | 0.688 | 0.514 |
|  | 1 | 0.819 | 0.925 | 0.442 |
|  | 1 | 1.287 | 1.243 | 0.265 |
| *CNA1* | 1 | 1.302 | 0.573 | 0.158 |
|  | 1 | 0.992 | 0.811 | 0.106 |
|  | 1 | 0.662 | 1.054 | 0.363 |
| *PLB1* | 1 | 1.109 | 8.259 | 0.218 |
|  | 1 | 1.725 | 7.341 | 0.556 |
|  | 1 | 1.247 | 6.580 | 0.345 |
| *PLB2* | 1 | 2.795 | 12.667 | 2.000 |
|  | 1 | 3.721 | 10.911 | 1.233 |
|  | 1 | 4.323 | 8.798 | 1.011 |
| *PLB3* | 1 | 1.981 | 1.564 | 0.284 |
|  | 1 | 2.291 | 1.112 | 0.764 |
|  | 1 | 3.613 | 1.747 | 0.384 |
| *PLB4* | 1 | 2.606 | 2.940 | 0.676 |
|  | 1 | 2.066 | 2.530 | 1.313 |
|  | 1 | 1.750 | 2.309 | 1.246 |
| *PLB5* | 1 | 2.032 | 5.282 | 1.192 |
|  | 1 | 1.925 | 3.283 | 1.341 |
|  | 1 | 1.110 | 3.792 | 0.736 |
| *PLC1* | 1 | 1.069 | 2.252 | 0.606 |
|  | 1 | 0.781 | 2.759 | 0.427 |
|  | 1 | 0.414 | 2.167 | 0.262 |

Abbreviation: FLC: fluconazole; BUD: budesonide.
